# Physicochemical Investigations on Samples Composed of a Mixture of Plant Extracts and Biopolymers in the Broad Context of Further Pharmaceutical Development

**DOI:** 10.3390/polym17111499

**Published:** 2025-05-28

**Authors:** Andreea Roxana Ungureanu, Adina Magdalena Musuc, Emma Adriana Ozon, Mihai Anastasescu, Irina Atkinson, Raul-Augustin Mitran, Adriana Rusu, Emanuela-Alice Luță, Carmen Lidia Chițescu, Cerasela Elena Gîrd

**Affiliations:** 1 Faculty of Pharmacy, “Carol Davila” University of Medicine and Pharmacy, 6 Traian Vuia Street, 020956 Bucharest, Romania; andreea.rxn@gmail.com (A.R.U.); emanuela.luta@umfcd.ro (E.-A.L.); cerasela.gird@umfcd.ro (C.E.G.); 2 Institute of Physical Chemistry—Ilie Murgulescu, Romanian Academy, 202 Splaiul Independenței, 060021 Bucharest, Romania; manastasescu@icf.ro (M.A.); iatkinson@icf.ro (I.A.); rmitran@icf.ro (R.-A.M.); arusu@icf.ro (A.R.); 3 Faculty of Medicine and Pharmacy, “Dunărea de Jos”, University of Galați, A.I. Cuza 35, 800010 Galați, Romania; carmen.chitescu@ugal.ro

**Keywords:** extracts’ mixture, PHB, PLGA, phytochemicals, ATR-FTIR, atomic force microscopy, stereomicroscopy, thermogravimetry

## Abstract

Vegetal sources are a continuous research field and different types of extracts have been obtained over time. The most challenging part is compounding them in a pharmaceutical product. This study aimed to integrate a mixture (EX) of four extracts (SE-*Sophorae flos*, GE-*Ginkgo bilobae folium*, ME-*Meliloti herba*, CE-*Calendulae flos*) in formulations with polymers (polyhydroxybutyrate, polylactic-co-glycolic acid) and their physicochemical profiling. The resulting samples consist of particle suspensions, which were subjected to Attenuated Total Reflectance Fourier Transform Infrared Spectroscopy analysis. When compared to single-extract formulations spectra, they revealed band changes, depending on the complex interactions. Using X-ray Diffractometry, the partially crystalline phase was highlighted for EX-PLGA, while the others were amorphous. Moreover, Atomic Force Microscopy pointed out the nanoscale particles and the topography of the samples, and the outstanding roughness belonging to EX-PHB-PLGA. A 30 min period of immersion was enough for the formulations to spread on the surface of the compression stockings material (CS) and after drying, it became a polymeric film. TGA analysis was performed, which evaluated the impregnated content: 5.9% CS-EX-PHB, 6.4% CS-EX-PLGA, and 7.5% CS-EX-PHB-PLGA. In conclusion, the extract’s phytochemicals and the interactions established with the polymers or with the other extracts from the mixture have a significant impact on the physicochemical properties of the obtained formulations, which are particularly important in pharmaceutical product development.

## 1. Introduction

Natural sources have been harnessed for remedies since ancient times. At first, the plant was used as a whole; then, the extraction processes evolved, resulting in the creation of the extracts, and advanced stages end with the isolation and purification of the active ingredients. The possibility of mixing different herbal remedies to obtain a synergistic effect was also outlined, and after association with different matrices, complex pharmaceutical products formed [1,2,3].

For chronic diseases, the most important challenge is to counteract the various destructive pathways, which simultaneously act in a degrading manner. A mixture composed of *Vitis vinifera* extract, *Ruscus aculeatus* extract, diosmetin and magnolog (from *Magnolia officinalis*) reduced inflammation in chronic venous disease by acting in several ways: hindering the release of cytokines and inhibiting NF-kB and AP-1 pathways [4]. A mixture of *Actaea racemosa* extract, *Oenothera biennis* extract, and *Hypericum perforatum* extract proved to be useful in climacteric symptomatology, each component being essential for polypharmacologic activity (antioxidant effect, stimulation of estrogen receptor, inhibition of DP1 receptor). Thus, a combination of extracts could be helpful in therapeutics due to its multi-targeted potential [5].

The benefits of using mixtures of extracts depend on the phytochemical composition of each extract. The most important advantages can be summarized as follows: 1. enhancing the same effect (e.g., antioxidant potential [6]); 2. development of complementary effects and 3. preventing antimicrobial resistance [7]. For example, the capillary protective effect which decreases capillary permeability reducing the formation of edema by extravasation is complementary to the veno-lymphatic drainer effect which, by stimulating lymphatic circulation, prevents lymph stagnation [8,9].

In the case of vascular chronic diseases, several useful plant sources are noted, including *Sophorae flos* for its rutin content with antioxidant, anti-inflammatory, and capillary-protective roles, *Ginkgo bilobae folium* for its flavonoid content with important properties for modulating peripheral circulation along with antioxidant and antiplatelet effect, *Meliloti herba* for its polyphenolic and coumarin complex with veno-lymphatic draining, capillary-protective and anti-inflammatory effects and *Calendulae flos* for its polyphenolic and saponin phytocomplex, useful in wound healing and sustaining microcirculation [10,11,12,13,14,15,16].

The use of polymers in pharmaceutical formulations aims to promote stability, increase bioavailability and optimize the release of active substances. Biopolymers, as polyhydroxybutyrate (PHB) and polylactic-co-glycolic acid (PLGA), are distinguished from classic polymers by their biodegradability, higher biocompatibility, lower toxicity, bioadhesive properties favoring tissue regeneration. They also play an important role in controlled and targeted drug delivery [17,18,19].

Besides therapeutic activity, the chemical composition of the extracts also influences their integration into pharmaceutical forms along with excipients, developing different types of interactions that leave their mark on the physicochemical properties of the new entities. Biopolymers can interact with active ingredients, such as phytocompounds from extracts, in several ways and purposes. One of the most common is forming adducts or complexes (through hydrogen bonds, electrostatic interactions [20]) favoring encapsulation for the purpose of controlled release or protection from degrading factors or increasing bioavailability (e.g., nanoformulations) [21,22]. Extracts also induce important changes to polymers depending on the type and concentration, being frequently used as additives to polymers for packaging films [23].

Most of the previous studies have focused on the inclusion in polymeric formulations of isolated phytochemicals (e.g., quercetin, catechin, bilobalide, ginkgolide [24,25]) or individual extracts (from one vegetal source [26,27] or from plant-mixtures [28]). The particularity of the current research derives from the inclusion of four extracts in PHB and PLGA polymeric formulations. Each extract or active substance can react differently in the presence of others; thus, a comparative analysis with the samples resulting in individual extracts is important in the context of a product with multiple components.

The current study is an extension of our previous approach that targeted the inclusion of individual extracts in formulations with biopolymers and the functionalization analysis of a compression stockings matrix [29]. In this preliminary stage, the purpose of the present study is to simultaneously integrate the extracts as a mixture (four extracts in equal parts) in biopolymeric formulations and characterize them in terms of physicochemical properties, also analyzing the impregnation on the compressive matrix.

## 2. Materials and Methods

### 2.1. Samples Preparation

Four dry vegetal extracts were obtained from four sources (*Calendulae flos*, *Ginkgo bilobae folium*, *Meliloti herba* and *Sophorae flos*) by ethanol reflux, rotary evaporation (Buchi, R-215) and lyophilization (Christ Alpha). The extracts’ phytochemical composition was determined by advanced chromatographic method (UHPLC-HRMS/MS). The results for three extracts (belonging from *Calendulae flos*, *Ginkgo bilobae folium* and *Sophorae flos*) were published in our previous work [30] and those for extract of *Meliloti herba* were provided in the Supplementary Materials.

The extracts’ mixture (EX) was obtained by mixing equal quantities of each extract (1:1:1:1, *w*/*w*) and homogenization. The equal ratio was chosen to exclude the dominance of one extract in order to conduct a comparative analysis (extracts’ mixture–polymer samples vs. extract–polymer samples).

Formulations were obtained by oil–water emulsification and solvent evaporation technique, as described in our previous work [29]. The oil phase was conducted with a polymer (PHB, PLGA and PHB:PLGA mixture 1:1 *w*/*w*) solution (0.5 g polymer/100 mL chloroform); the water phase consists of a mixture of extracts dissolved in ethanol; the two phases were mixed (700 rpm, 5 min, magnetic stirring) and added to 100 mL of 3% polyvinyl alcohol dispersion in water (2 min, ice bath), then placed under a magnetic stirrer (1000 rpm, 2 h) for solvent evaporation [29,31].

### 2.2. Attenuated Total Reflectance Fourier Transform Infrared Spectroscopy (ATR-FTIR)

The ATR-FTIR spectra were recorded by a JASCO FTIR-4200 spectrophotometer (equipped with ATR-PRO450-S accessory, Tokyo, Japan) under the following wavenumbers range: 4000–400 cm^−1^ (single bonds region 4000–2500 cm^−1^, triple bonds region 2500–2000 cm^−1^, double bonds region 2000–1500 cm^−1^ and fingerprint area < 1500 cm^−1^) at a resolution of 4 cm^−1^ [29,32,33,34].

### 2.3. X-Ray Diffractometry (XRD)

Rigaku Ultima IV diffractometer (Rigaku Co., Tokyo, Japan) was used for XRD analysis under the following parameters: parallel beam geometry, CuKα radiation (λ = 1.5406 Å), 1.00 mm (divergent slit size), 10 mm (divergent height limiting slit), interval of 10–60° at 2°/min speed, step size of 0.02°, 2θ range. The diffractograms were recorded on solid samples (thin polymeric films) obtained by drying at room temperature (20 ± 5 °C, 24 h) [29,35].

### 2.4. Atomic Force Microscopy (AFM)

XE-100 atomic force microscope (Park Systems Corporate, Suwon, Republic of Korea) with decoupled XY/Z scanners and NSC36B tips (MikroMasch, Sofia, Bulgaria), was used for the AFM analysis. The tip was operating in non-contact mode and is described by the following dimensions: thickness of 1 μm, height of 15 μm, length of 90 μm, width of 32 μm, less than 8 nm radius curvature, full cone angle: 40°, resonance frequency: 130 kHz. XEI program (v 1.8.0, Park Systems Corporate, Suwon, Republic of Korea) processed the AFM recorders in enhanced-contrast mode. The topographic images were captured at two scales (8 × 8) µm^2^ and (2 × 2) µm^2^ [29,36,37,38].

### 2.5. Extensibility Assay and Matrix Material Impregnation Analysis

The spreading ability of the samples was assessed using an extensiometric device (two overlapping plates, the lower plate being millimeter graduated on its external side). The sample (0.5 g) was exposed to additional weights (first the weight of the upper plate: 150 g, then 100 g, 200 g, 300 g, 400 g, 500 g and 600 g were added) every 1 min. The results are presented as an extension surface [29,39].

In addition to the extensibility determination, the impregnation on textile matrix type material was evaluated. Considering that the extracts used have a role in the treatment of vascular diseases, application on the material of compression stockings was attempted by immersing the matrix (2 × 2 cm^2^ pieces) in samples and keeping it in contact for different times (30 min, 60 min and 90 min). The impregnated material was weighed before and after drying and analyzed under a stereomicroscope (Zeiss Stemi 508 Greenough StereoMicroscope, 8:1 zoom, 50× magnification, Oberkochen, Germany).

### 2.6. Thermogravimetric Analyses

The thermogravimetric analysis (TG) and the derivative thermogravimetric analysis (DTG) underwent the impregnated matrices (pieces of compression stockings, 2 × 2 cm^2^) based on a Mettler Toledo TGA/SDTA851e thermogravimeter (Mettler Toledo, Greifensee, Switzerland), performed at 10 °C/min heating rate and synthetic air flow, under 80 mL/min [29,36].

### 2.7. Statistical Analysis

The extensibility and impregnation assay results are stated as the mean ± SD (standard deviation of the mean of *n* = 6). The error bars are included in the figures.

## 3. Results

### 3.1. Samples Preparation

**Figure 1:**
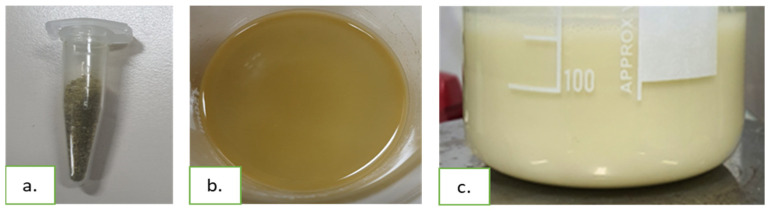
Preparation of samples with vegetal extracts’ mixture and biopolymers: (**a**) mixture of vegetal extracts as dry powder; (**b**) mixture of extracts dissolved in ethanol; (**c**) formulation with vegetal extracts and polymers.

The extracts’ mixture consists of a yellowish-green powder, determined by the colors of the mixed extracts, and when dissolved in ethanol, it shows a yellow-brown color determined by the solubilization of the phytoconstituents. The resulting samples were suspension-type formulations, due to the use of PHB and PLGA polymers known as particle formers. The particles are suspended in the polyvinyl alcohol dispersion, giving a macroscopically homogenous appearance (Figure 1).

### 3.2. Attenuated Total Reflectance Fourier Transform Infrared Spectroscopy (ATR-FTIR)

**Figure 2:**
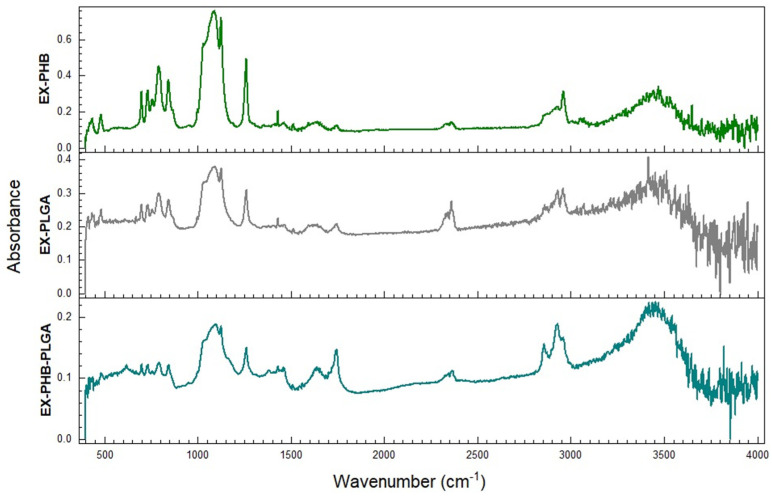
ATR–FTIR spectrograms (EX = mixture of extracts, PHB = polyhydroxybutyrate, PLGA = polylactic-co-glycolic acid).

ATR-FTIR spectra were presented in Figure 2. All the samples show wide bands in the single bond region (4000–2500 cm^−1^), especially the region of stretch vibrations of O-H bonds, revealing peaks at 3483.9 cm^−1^ for EX-PHB, 3458.6 cm^−1^ for EX-PLGA and 3430.1 cm^−1^ for EX-PHB-PLGA. Bands corresponding to vibrations of the C-H bonds were also observed. In the region 2970–2950 cm^−1^, attributed to the asymmetric stretching vibration of C-H bonds from methyl groups, EX-PHB peak at 2958.3 cm^−1^ and the other two samples, EX-PHB-PLGA and EX-PLGA, peak at the same value of 2952 cm^−1^. Regarding the region 2935–2915 cm^−1^, attributed to the asymmetric stretching vibration of C-H bonds from methylene groups, EX-PHB peak at 2926.6 cm^−1^, EX-PLGA peak at 2929.8 cm^−1^, and EX-PHB-PLGA peak at a higher value of 2933 cm^−1^. Also, for EX-PHB-PLGA, a peak at 2857 cm^−1^ was observed, in the region of symmetric stretching vibrations of C-H from methylene groups (2865–2845 cm^−1^).

Other distinctive peaks were found in the double bond region (2000–1500 cm^−1^): 1745.6 cm^−1^ for EX-PHB, 1739.3 cm^−1^ and 1725 cm^−1^ for EX-PLGA, 1742.5 cm^−1^ for EX-PHB-PLGA, attributed to stretching vibrations of C=O bonds (1780–1650 cm^−1^); 1650.7 cm^−1^ for EX-PHB, 1622.2 cm^−1^ for EX-PLGA and 1634.8 cm^−1^ for EX-PHB-PLGA, attributed conjugated quinones or ketones (1650–1600 cm^−1^) or to C=C bonds from alkenyl groups (1680–1620 cm^−1^). Peaks attributed to aromatic rings were found for EX-PHB (1508.2 cm^−1^) and for EX-PLGA (1520.8 cm^−1^). The lack of aromatic ring’s peak for the sample with a mixture of extracts and mixture of polymers (PHB-PLGA) may be determined by a masking effect given by the height of the peak at 1634.8 cm^−1^, or extension of conjugation resulting in a complex.

### 3.3. X-Ray Diffractometry (XRD)

**Figure 3:**
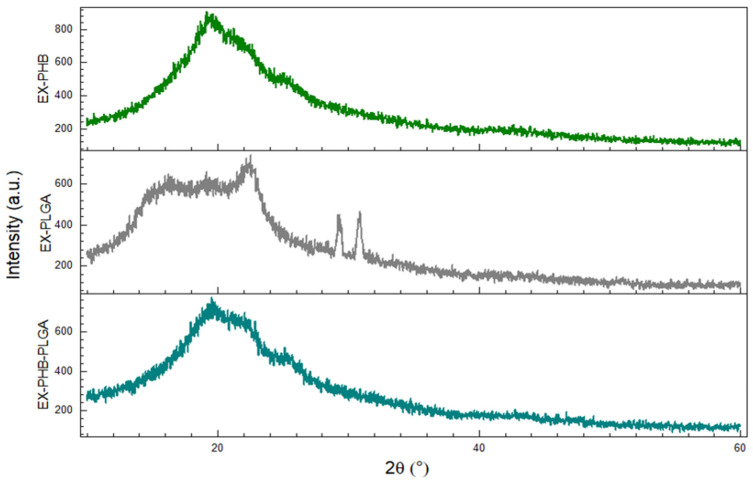
Diffractograms of extracts’ mixture and polymer formulations (EX = mixture of extracts, PHB = polyhydroxybutyrate, PLGA = polylactic-co-glycolic acid).

Among the samples with extracts’ mixture (Figure 3), EX-PHB and EX-PHB-PLGA are amorphous. Although EX-PLGA seems amorphous at first, several peaks can be noticed, imprinting partially crystalline structure (22.5°, 742 a.u.; 29.34°, 454 a.u.; 30.86°, 469 a.u.). It is possible that, in the case of this formulation, crystallinity is given by the ME. The phytocompounds in its composition, which are in crystallized form, have a more stable structure and can establish fewer bonds with the components of the mixture, allowing the amorphous part of ME to interact.

### 3.4. Atomic Force Microscopy (AFM)

**Figure 4:**
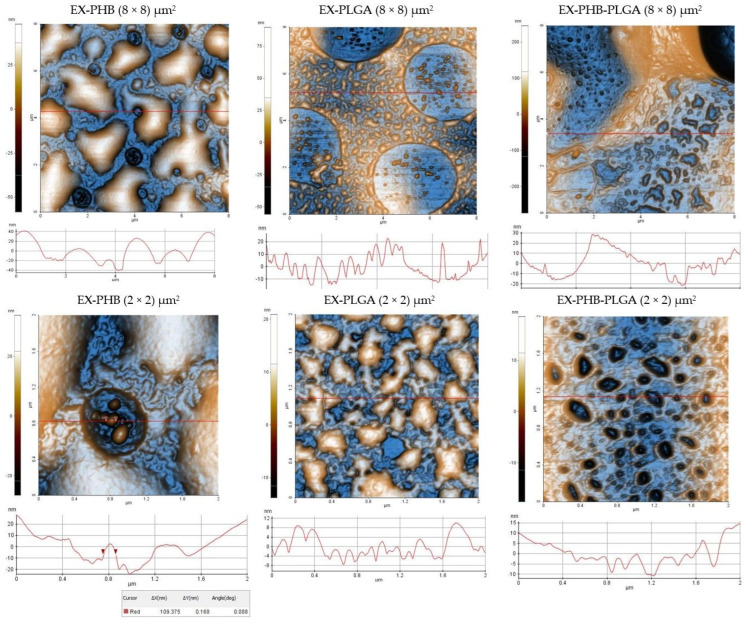
AFM topographic 2D images with surface profiles (red lines) of extracts’ mixture–polymer samples (EX = mixture of extracts, PHB = polyhydroxybutyrate, PLGA = polylactic-co-glycolic acid).

The analysis was carried out at two scales (8 × 8) µm^2^ and (2 × 2) µm^2^, resulting in 2D and 3D high-contrast topographic images. Two-dimensional images are presented in Figure 4 and 3D images and results for samples with *Meliloti herba* extract are presented in the Supplementary Materials.

At the higher scale, (8 × 8) µm^2^, the EX-PHB sample presents large nanostructures with undefined shapes, which may be the result of aggregation, nanopores and small nanoparticles. For the EX-PLGA sample, irregularly shaped aggregates are observed, but smaller in comparison with EX-PHB; its pores are wider and they are delimited by the formation of aggregates. The EX-PHB-PLGA sample has a distinct appearance by the presence of cavities, some larger, others smaller; nanoparticles and aggregates with a filiform appearance are also observed.

In-depth analysis, at (2 × 2) µm^2^ scale, revealed significant differences between the samples. EX-PHB presents large particle aggregates (the order of micrometers), small nanoparticles (<100 nm) and large pores. EX-PLGA has a more uniform appearance, characterized by aggregates with an undefined shape, but smaller than for EX-PHB and small pores with high density. EX-PHB-PLGA also has a uniform appearance, based on smaller and more dispersedly distributed nanostructures which generate large regions of separation between nanostructures (large pores). For EX-PLGA, uniformity is given by large nanostructures with high density, revealing small pores. The appearance of EX-PHB at (8 × 8) µm^2^ is similar to EX-PLGA at (2 × 2) µm^2^, suggesting that the EX-PLGA sample has a smoother surface.

Topographic images descriptions are complemented by roughness parameters (Rpv = peak to valley, Rq = root mean square roughness, Ra = average roughness). Data are presented in Table 1.

At 8 × 8 μm^2^, significant differences can be observed between Rq values. Regarding the whole surface, EX-PHB-PLGA showed the highest value, followed by EX-PHB (approximately three times lower) and EX-PLGA. More detailed analysis by the profile line, revealed a higher Rq value for EX-PHB, the lowest value for EX-PLGA and EX-PHB-PLGA sample’s value being intermediate.

More in-depth analysis, at 2 × 2 μm^2^ scale, showed that the EX-PHB sample stands out, recording the highest Rq value for the whole surface and for the profile line (red line). The root mean square roughness is approximately two times higher for EX-PHB than the other two samples, EX-PLGA and EX-PHB-PLGA, which presented similar values.

The two-scale analysis performed provided a complex perspective on the formulations obtained. The higher scale allowed the examination of the characteristics on an extensive surface, while the lower scale allowed an in-depth analysis.

### 3.5. Extensibility Assay and Matrix Material Immersion Analysis

**Figure 5:**
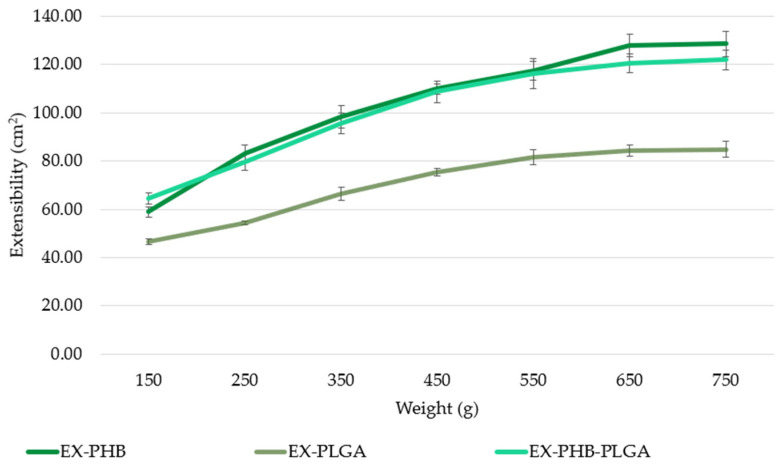
Extensibility assay results (EX = mixture of extracts, PHB = polyhydroxybutyrate, PLGA = polylactic-co-glycolic acid).

Extensibility measurement results were shown in Figure 5. The variation follows the succession: EX-PHB (128.65 ± 5.29 cm^2^) > EX-PHB-PLGA (122.03 ± 4.09 cm^2^) > EX-PLGA (84.93 ± 3.27 cm^2^). The extensibility of the sample with both polymers proved to be close to the sample with PHB.

All samples had high extensibility, being suitable for display on skin and on a solid matrix (e.g., compression stockings material).

**Figure 6:**
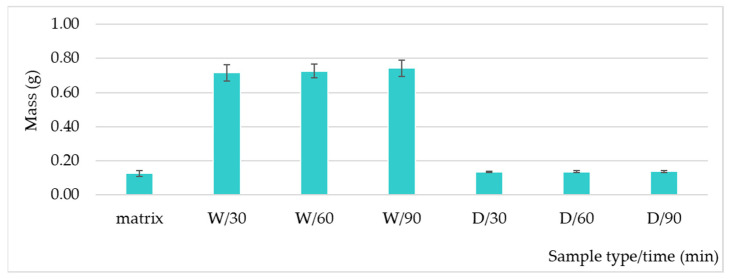
Mass variation for compressive material samples immersed in formulations (W = wet sample, immediately after immersion, D = dry sample, after 12 h room temperature rest).

The compressive matrix impregnation analysis led to the results presented in Figure 6. After the immersion of compression matrix (2 × 2 cm^2^ pieces) in formulations, for wet samples, a mass increase of about five times the initial mass (0.1255 g ± 0.0165 g) was observed in the first 30 min. It can be determined by rapid wetting of the material as well as particle impregnation. A very slight subsequent increase in mass was noticed for wet samples: 30 min (0.7151 g ± 0.0462 g) < 60 min (0.7258 g ± 0.0390 g) ≅ 90 min (0.7425 g ± 0.0488 g). After drying the samples, no significant differences in mass were noticed between immersion times: 30 min (0.1366 g ± 0.0038 g) ≅ 60 min (0.1367 g ± 0.0044 g) ≅ 90 min (0.1369 g ± 0.0039 g); therefore, immersion for a period longer than 30 min does not have important advantages.

**Figure 7:**
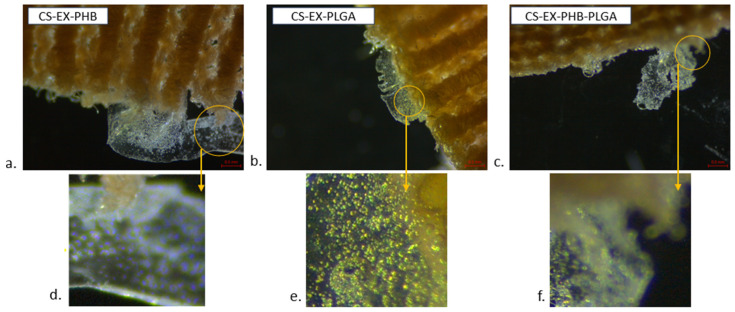
Stereomicroscopic images of compression stockings matrix immersed in formulation after 12 h drying at room temperature (brown mass = compression stockings material; translucent mass = thin polymeric film with extracts’ mixture–polymer particles, CS = compression stockings material, EX = mixture of extracts, PHB = polyhydroxybutyrate, PLGA = polylactic-co-glycolic acid, (**a**–**c**) = overview stereomicroscopic images, (**d**–**f**) = magnified view of the region marked in (**a**–**c**)).

The dried samples (stereomicroscopy analyzed) displayed on their surface a polymer film, formed after immersion in formulations and drying (Figure 7).

### 3.6. Thermogravimetric Analysis

**Figure 8:**
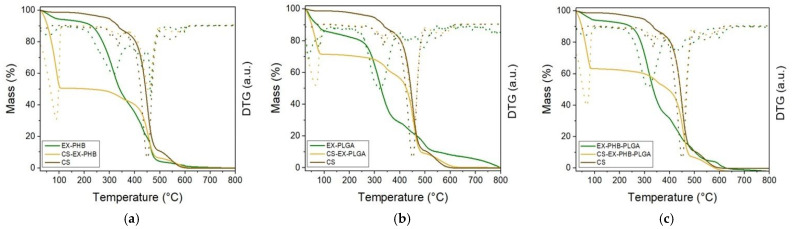
Thermogravimetric curves (EX = mixture of extracts, PHB = polyhydroxybutyrate, PLGA = polylactic-co-glycolic acid, CS = compression stockings material, full lines = TGA results by thermogravimetry, dotted lines = DTG results by derivative thermogravimetry, (**a**) = thermogravimetric curve for EX-PHB sample, (**b**) = thermogravimetric curve for EX-PLGA sample, (**c**) = thermogravimetric curve for EX-PHB-PLGA sample).

Decomposition behavior and thermal stability were assessed by thermogravimetry; the results are illustrated in Figure 8 and for ME samples, in the Supplementary Materials.

For compression stockings material, the mass loss events occurred at three intervals: the first was between 280 °C and 380 °C, the second was between 380 °C and 500 °C (highest mass loss) and the third was between 500 °C and 620 °C [29].

For the extracts’ mixture–polymer formulations (EX-PHB, EX-PHB-PLGA and EX-PLGA), also three main mass-loss intervals described the thermogravimetric curve. The first is attributed to the loss of volatile compounds (25–115 °C), recording a gradual mass-loss event. The second, corresponding to organic substances combustion, marked the most important mass loss (from 10–15% to 85–90%) between 250 °C and 500 °C. The third interval (500–800 °C), following a gentle downward slope, can be attributed to the oxidation of carbon residues (resulted from polymers and extracts’ phytochemicals degradation) [40,41].

**Figure 9:**
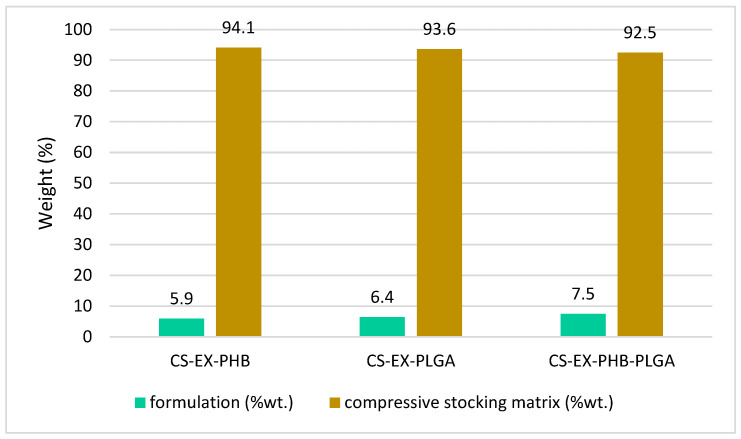
Impregnation results from thermogravimetric analyses (EX = mixture of extracts, PHB = polyhydroxybutyrate, PLGA = polylactic-co-glycolic acid, CS = compression stockings material).

For the compression stockings’ material impregnated with formulations, the matrix material is the principal component for overall mass, suggested by the thermal behavior of the sample which was similar to the matrix alone. In the first stage (<115 °C), significant loss of volatile compounds was noted for all samples; it was remarkable for CS-EX-PHB (50%), followed by CS-EX-PHB-PLGA (35%) and CS-EX-PLGA (30%). In the second temperature interval, 115–380 °C, the sample mass remained constant (a plateau). In the third interval, 380–480 °C, the highest weight loss was noticed (90%). The amount of extracts’ mixture–polymer formulation impregnated on the compression stockings matrix was computed from the mass loss values at 380 °C, related to the mass of the dry sample at 115 °C (Figure 9).

All samples had impregnation above 5%, with the best results for EX-PHB-PLGA formulation, 7.5%.

## 4. Discussion

Within this work, three samples of extracts’ mixture–polymer formulation were obtained from four vegetal extracts and two polymers. The extracts, due to their phytocomplex, represent the main pharmacologically active component. Our previous research found that SE is rich in flavonoids (rutin > isorhamnetin > quercetin > hesperetin > genistein). GE is also rich in flavonoids, but contains a significant amount of gallic acid (isorhamnetin > quercetin > rutin > gallic acid > hyperoside) [30]. ME contains flavonoids and high amounts of coumaric acid and chlorogenic acid (rutin > hyperoside > epicatechin > coumaric acid > chlorogenic acid) (Supplementary Materials of the current study). CE is distinguished by chlorogenic acid quantity followed by flavonoid content and syringic acid (chlorogenic acid > isorhamnetin > rutin > hyperoside > syringic acid) [30]. Even though the polymers are adjuvant components, PHB and PLGA have an essential role because they are nanoparticle-forming agents. Both PHB and PLGA have very good biocompatibility. Regarding encapsulation, PHB encapsulates better lipophilic compounds, while PLGA is more versatile, encapsulating both lipophilic and hydrophilic substances equally well. The association of the extracts with the two biopolymers can lead to advanced nanosystems improving the bioavailability of active phytochemicals [42,43].

Formulations were obtained by oil–water emulsification and solvent evaporation and underwent to ATR-FTIR analysis. The resulting ATR-FTIR spectra were compared with those of the extracts, polymers and the extract–polymer formulations which have been presented in our previous work (for SE, GE, CE) [29] and in Supplementary Materials (for ME).

In the single bonds area (4000–2500 cm^−1^), in the region of stretching vibrations of O-H alcoholic group, the most pronounced peak was noticed for EX-PHB (3483.9 cm^−1^), highlighting the most significant shift to a higher wavenumber in comparison with main components spectra: extracts and polymer (SE: 3407.9 cm^−1^, GE: 3398.4 cm^−1^, ME: 3395.3 cm^−1^, CE: 3401.6 cm^−1^, PHB: 3430.1 cm^−1^ [29], ME: 3395.3 cm^−1^ (Supplementary Materials)) and also in comparison with individual extract–polymer samples (SE-PHB: 3461.7 cm^−1^, GE-PHB: 3401.6 cm^−1^, CE-PHB: 3436.4 cm^−1^ [29], ME-PHB: 3445.9 cm^−1^ (Supplementary Materials)). Thus, PHB in combination with vegetal extracts’ mixture led to weak hydrogen bonds, less associated O-H groups which vibrate easier, conducting to a higher wavenumber shift.

For the EX-PLGA sample, the most pronounced peak was at 3458.6 cm^−1^, being a higher wavenumber than for extracts and extract–polymer samples (SE-PLGA: 3433.2 cm^−1^ [29], ME-PLGA: 3447.4 cm^−1^ (Supplementary Materials), CE-PLGA: 3455.4 cm^−1^ [29]), but lower than the value for polymer (PLGA: 3645.4 cm^−1^ [29]) and GE-PLGA sample (3488.8 cm^−1^ [29]). Thus, the hydrogen bonds from EX-PLGA sample can be considered weaker (high wavenumber) than those for individual extract–polymer samples. Regarding the difference between the GE-PLGA sample and EX-PLGA, it is possible that the association of GE with the other extracts to facilitate bonding of GE with polymer in EX-PLGA, resulting in lowering the wavenumber (stronger bonds). Competition for hydrogen bonding may arise due to the complexity of the mixture. A study on the development of a drug delivery system with carmofur revealed a high encapsulation capacity due to strong hydrogen bonds [44]. The same was observed for a system with flavonoids (quercetin, luteolin, resveratrol, and curcumin) in quinoa protein nanomicelles [45]. Weakening of hydrogen bonds (such as pH change) led to faster release of the drug content through destabilization (swell and dissociation) [44]. Even though this could be an advantage for the release of the active compounds, it may constitute a disadvantage for stability.

For the EX-PHB-PLGA sample, principal peak (single bonds region) is at 3430.1 cm^−1^, value superior to extracts, identical with PHB and inferior to PLGA and individual extract–polymers samples (SE-PHB-PLGA: 3449.1 cm^−1^ [29], CE-PHB-PLGA: 3433.2 cm^−1^ [29], ME-PHB-PLGA (Supplementary Materials) and GE-PHB-PLGA: 3468.1 cm^−1^ [29]). In this case, it could be considered that interactions between EX-PHB-PLGA samples are predominantly determined by PLGA and extracts’ mixture, PHB being less involved because overall, the sample has the same peak as PHB. Competition between polymers may occur.

Regarding XRD analysis, according to our previously published results, three extracts (SE, GE, CE) and PLGA were in the amorphous phase and PHB was in crystalline state [29]. A notable aspect was the partially crystalline phase for ME (results in the Supplementary Materials), it showed a small peak at 30°, 900 a.u.). This can be attributed to its chemical composition rich in phenolic acids (coumaric acid and chlorogenic acid), which, having a simpler structure, allow them to be arranged more easily in a crystal [46]. However, for the individual extract–polymer formulations (ME-PHB, ME-PLGA, ME-PHB-PLGA), the obtained samples are amorphous (Supplementary Materials). When mixed with other extracts, competition may occur for interactions with the polymer and it is possible that the other extracts (SE, CE, GE) disrupt the interaction ME-polymer.

Related to AFM conducted analysis, the samples with a mixture of extracts had a complex topographic appearance, characterized by the formation of large aggregates, comparatively with one-extract samples which showed smaller aggregates (Supplementary Materials and previously published work [29]). These can be determined by the technique of obtaining when the solvent-evaporation leads to the proximity of the suspended particles causing aggregates [47]. In addition, in the case of samples consisting of the extracts’ mixture, each extract contributes with a certain quantitative content of phytocompounds; for example, flavones. Thus, it can be considered that the mixture contains a greater amount of flavones than the extracts taken individually. Adsorption may occur on the surface of the particles [48,49]. Flavones have ionizable groups in their structure [50]; for example, quercetin is a pentahydroxyflavone with five ionizable hydroxyl groups [51]. Due to these groups, these compounds can alter the surface charge of the polymeric particles affecting colloidal stability.

The topographic aspect can be influenced by the properties of the polymers and the way in which they interact with the extract mixture. The structural reorganization of PHB is more difficult (PHB is crystalline), which could explain the formation of larger nanoparticles, while PLGA (being amorphous) allows for faster self-assembly, which can lead to smaller nanoparticles. PHB can be considered more hydrophobic than PLGA (has terminal carboxyl group from glycolic acid). For PHB, studies have noted the formation of small spherical nanoparticles (around 250 nm), which increase their size when incorporating active compounds (for curcumin the size doubles, around 500 nm), aggregates can also result [52,53,54,55]. In the aqueous medium (in our case aqueous dispersion of polyvinyl alcohol), the colloidal stability of hydrophobic polymers is affected, favoring interactions between nanoparticles. The larger their dimensions, the more easily aggregates are formed. According to a study on silica nanoparticles, those below 190 nm have reduced aggregation due to the influence of hydration with accentuated electrostatic repulsion. The particles above 190 nm have more pronounced aggregation because the influence of hydration decreases and attractions become more pronounced [56]. This could also explain the visibility of the pores: small nanoparticles are more uniform distributed on the surface, with the large pores not being covered by aggregates as in the case of large nanoparticles.

In our current research and development stage for a complex pharmaceutical product, consisting in nanoformulation and a support matrix (as compression stockings), the preliminary analyses of matrix impregnation were as follows: extensibility assay, weighing of immersed material and stereomicroscopy evaluation.

In terms of extensibility, regarding the mixture of extracts related to polymers: some extracts can be plasticizers (e.g., *Centella asiatica* extract and gelatin [23]), others can be stiffeners (e.g., berries extract and gelatin [23]); however, the possibility that an extract is plasticizing with a certain polymer and stiffening with another should not be neglected. These behaviors affect extensibility in a more complex way in the case of extracts’ mixture. The formulations showed adequate extensibility for the extension on the compressive stockings’ material. After 30 min immersion and drying for 12 h at room temperature, the formation of a thin polymeric layer on its surface was observed (stereomicroscopic observation).

The samples were subjected to thermogravimetric analyses to assess their thermal stability and for an in-depth impregnation study. Within the large intervals of decomposition, several superimposed events were noticed (DTG broad inverted peaks) which can be determined by the same decomposition intervals of organic phytocompounds from extracts and polymers [57,58,59,60]. For PHB, decomposition starts slowly around 200 °C and accelerates above 260 °C, by polymeric chains degradation (resulting in crotonic acid, dimeric, trimeric and tetrameric volatile compounds) [57,58]. PLGA significant decomposition begins (around 170 °C) by breaking the bond between lactic and glycolic acid (first a random chain scission and specific chain scission at the end) [61] and continues to 370 °C (with significant decomposition at 275 °C), resulting in CO_2_ (terminal carboxyl groups decarboxylation) [61]. For PLGA nanoparticles, the main thermal degradation processes can be in the interval 300–400 °C [59]. Flavonoids are the most abundant phytocompounds in our extracts. In the air atmosphere, quercetin has three thermal decomposition stages (103–342 °C, 342–428 °C and 428–605 °C) and rutin has four (50–117 °C, 117–261 °C, 261–422 °C, 422–604 °C) [61]. Thus, for our extracts’ mixture–polymer formulations, the interval 200–500 °C is the most susceptible to superposed events, which can explain the highest mass loss.

Another important aspect for superposed events is that extracts have antioxidant activity, imprinted by polyphenolic content [30], acting as stabilizers. At the same time, the extracts can enhance polymer’s degradation by their decomposition products. A previously reported study revealed that rosemary extract and green tea extract slightly reduced the thermal stability of a PHB derivate polymer [62].

The impregnation study derived from the thermogravimetry data, compared to the results for one-extract samples (SE, GE, CE previously published [29] and ME presented in Supplementary Materials), revealed that CS-EX-polymer samples are intermediate impregnated. Regarding the active component of the formulation, the following successions were noticed: ME < EX < GE < SE < CE (PHB samples); GE < SE < EX < CE < ME (PLGA samples); GE < CE < EX < SE < ME (PHB-PLGA samples). For example, in PHB-PLGA succession (the most complex samples), GE’s and CE’s samples showed low impregnation. The most important factor was the sample’s topography, which is dependent on the established interactions between the extract and the polymer, leading to specific physicochemical characteristics of the formulation. SE’s and ME’s samples revealed high impregnation. Thus, the extracts’ mixture sample led to an intermediate impregnation.

Among the physicochemical properties of formulations, nanoparticle dimensions are a significant factor for impregnation, influencing the specific surface, stability, and penetration into the compression stockings material. Smaller particles can deeply penetrate the material and probably lead to a high impregnation, but this is not an advantage for release at the skin level. The presence of pores (visualized through AFM analysis) increases the contact surface, but if the dimensions are large, can cause gaps in the polymeric film concomitant with uneven distribution on compression stockings matrix. Also, another important factor is aggregation, which reduces stability and specific surface.

Stability analysis of the nanoformulations is a future research perspective, along with optimization studies, in order to acquire less variable physicochemical properties that would allow reproductibility. In vitro release studies for active phytochemicals represent a further direction of analysis, as well as the study of transcutaneous permeability.

## 5. Conclusions

Within this study, a mixture of extracts was prepared from four dry extracts belonging to four natural sources (*Sophorae flos*, *Ginkgo bilobae folium*, *Meliloti herba* and *Caledulae flos*) and was integrated into polymeric formulations with polyhydroxybutyrate and polylactic-co-glycolic acid. The ATR-FTIR results allowed comparative analysis with the samples of individual extracts, concluding that the complexity of the samples leads to competition for hydrogen bonding. XRD analysis revealed that the EX-PLGA sample is partially crystalline. AFM images captured the topography of the samples, confirming that the obtained formulations are in nanoscale and emphasized the aggregations which are important for further stability studies. The samples showed adequate extensibility and after drying, the immersed compression stockings material, a thin polymeric film was observed on its surface. The degradation profiles were outlined (by TGA) and the material impregnation was analyzed, with the best results produced by CS-EX-PHB-PLGA, followed by CS-EX-PLGA and CS-EX-PHB. While these findings are promising, contributing to a preliminarily understanding of the physicochemical complexity of extracts’ mixture–polymer samples, further research is necessary in terms of stability, optimization and therapeutic efficacy.

## Figures and Tables

**Table 1:** Roughness parameters for extracts’ mixture formulations (Rpv = peak to valley, Rq = root mean square roughness, Ra = average roughness, EX = mixture of extracts, PHB = polyhydroxybutyrate, PLGA = polylactic-co-glycolic acid).

Scale	8 × 8 μm^2^						2 × 2 μm^2^					
	Whole Surface			Red Line Profile			Whole Surface			Red Line Profile		
Parameter (nm)	Rpv	Rq	Ra	Rpv	Rq	Ra	Rpv	Rq	Ra	Rpv	Rq	Ra
EX-PHB	106.65	19.185	16.045	81.926	21.996	18.263	60.335	11.078	9.078	52.201	12.128	9.841
EX-PLGA	145.68	17.703	13.45	37.607	9.153	7.573	34.701	5.898	4.842	17.58	4.374	3.6
EX-PHB-PLGA	519.144	59.758	32.872	49.723	12.129	9.616	36.116	5.829	4.752	25.295	5.83	4.7

## Data Availability

Data are contained within the article and Supplementary Materials.

## Supplementary Materials

The following supporting information can be downloaded at: https://www.mdpi.com/article/10.3390/polym17111499/s1, Figure S1: AFM 3D enhanced contrast images for EX samples; Figure S2: AFM 3D enhanced contrast images for ME samples; Figure S3: AFM 2D enhanced contrast images for ME samples with profile lines; Figure S4: ATR-FTIR comparative spectrograms for ME samples; Figure S5: XRD comparative results for ME samples; Figure S6: Thermogravimetric results for ME samples; Table S1: Quantitative UHPLC-HRMS/MS results for *Meliloti herba* extract (ME). Table S2: Roughness parameters for ME samples; Table S3: ATR-FTIR comparative analysis for ME samples; Table S4: The content of formulation impregnated on the compression stockings matrix estimated by thermogravimetry for ME samples.
