# Supplementary material for: Physicochemical Investigations on Samples Composed of a Mixture of Plant Extracts and Biopolymers in the Broad Context of Further Pharmaceutical Development
Source: Polymers (Basel). 2025 May 28;17(11):1499. doi: 10.3390/polym17111499 (PMC12157323; doi:10.3390/polym17111499)
Supplement: Supplementary file 1 [file polymers-17-01499-s001.zip › polymers-3624740-supplementary.pdf]

Article

Physicochemical Investigations on Samples Composed of a Mixture of Plant Extracts and Biopolymers in the Broad Context of Further Pharmaceutical Development

Supplementary Materials

Table S1. Quantitative UHPLC-HRMS/MS results for *Meliloti herba* extract (ME).

Cuantified compound	Phytochemical class	Amount (μg/g)
apigenin	flavonoid	9.18
chrisin	flavonoid	122.97
daidzein	flavonoid	29.58
epicatechin	flavonoid	854.8
formononetin	flavonoid	10.71
galangin	flavonoid	254.04
genistin	flavonoid	/
glycytein	flavonoid	/
hesperetin	flavonoid	/
hyperosid	flavonoid	1318.87
isorhamnetin	flavonoid	/
kaempferol	flavonoid	/
naringenin	flavonoid	3.07
pinocembrin	flavonoid	4.1
quercetin	flavonoid	/
rutin	flavonoid	5233.24
acid cafeic	phenolic acid	/
chlorogenic acid	phenolic acid	327.89
ferulic acid	phenolic acid	294.24
gallic acid	phenolic acid	171.77
p-coumaric acid	phenolic acid	641.99
syringic acid	phenolic acid	/
abscisic acid	sesquiterpen	187.42

3D	EX-PHB	EX-PLGA	EX-PHB-PLGA
8 µm x 8 µm	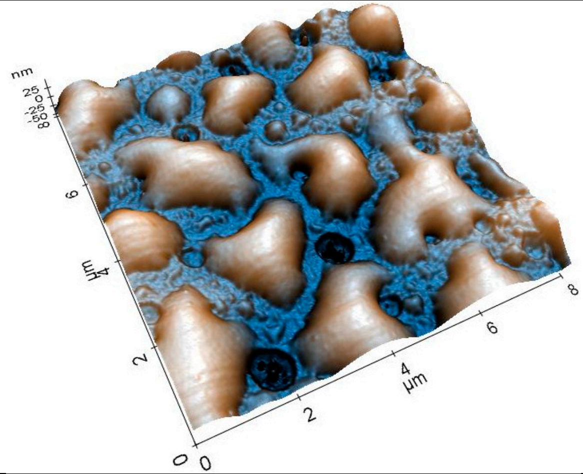	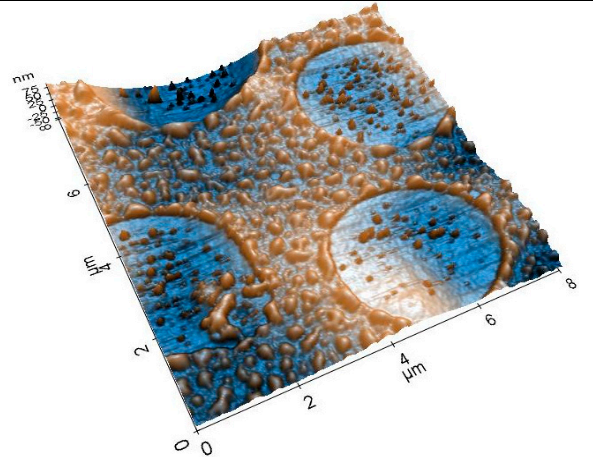	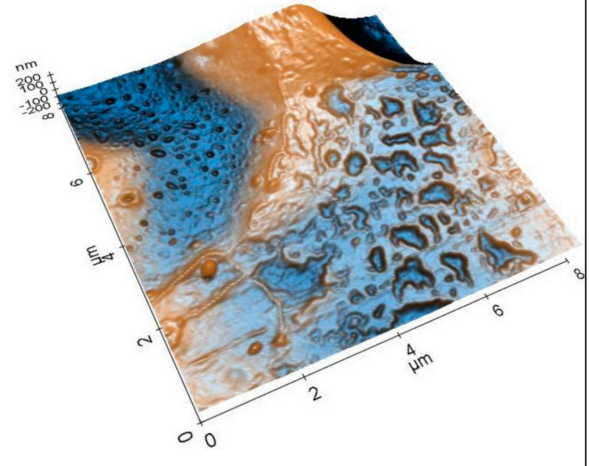
2 µm x 2 µm	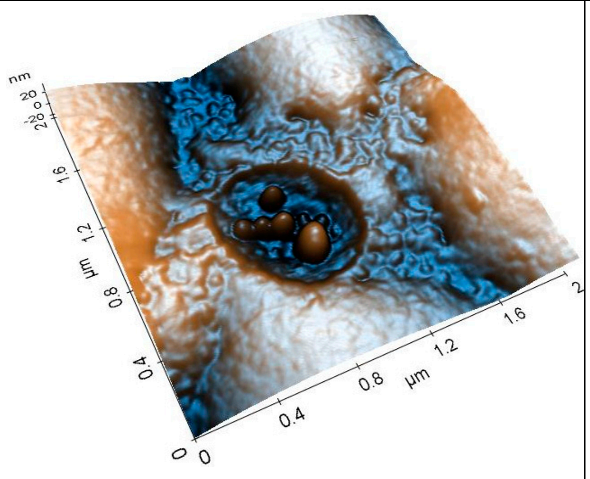	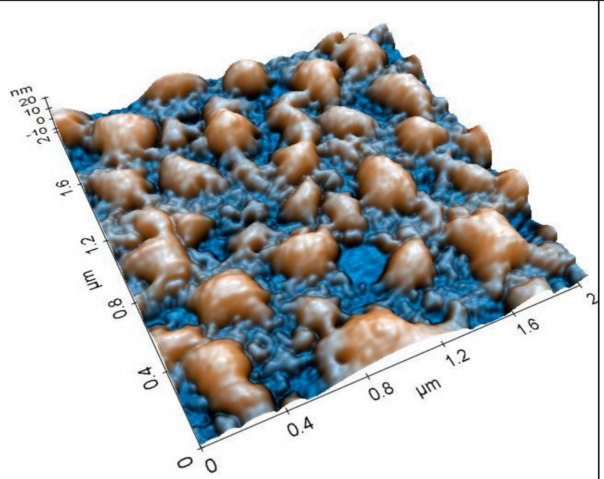	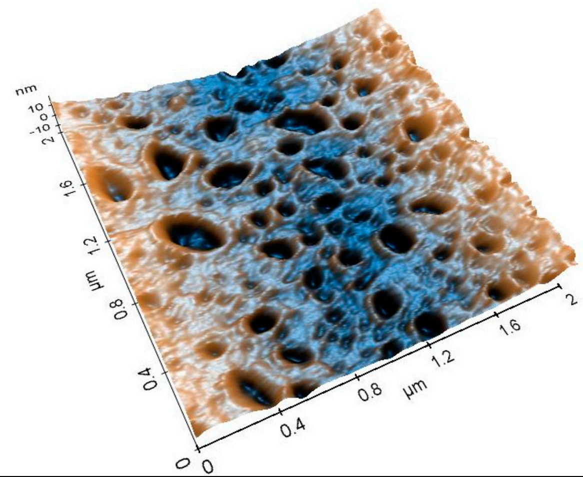

Figure S1. AFM 3D enhanced contrast images for EX samples (EX=mixture of extracts, PHB = polyhydroxybutyrate, PLGA = polylactic-co-glycolic acid).

3D	ME-PHB	ME-PLGA	ME-PHB-PLGA
8 µm x 8 µm	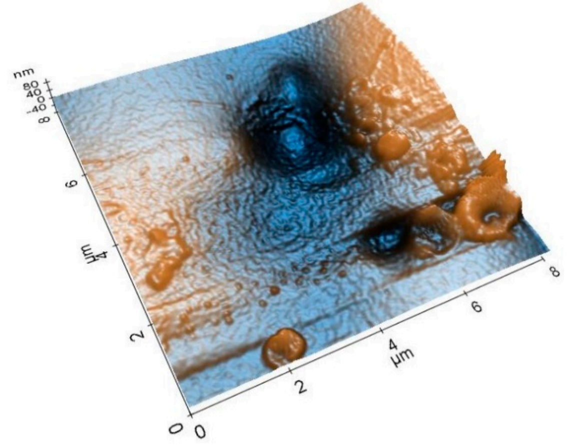	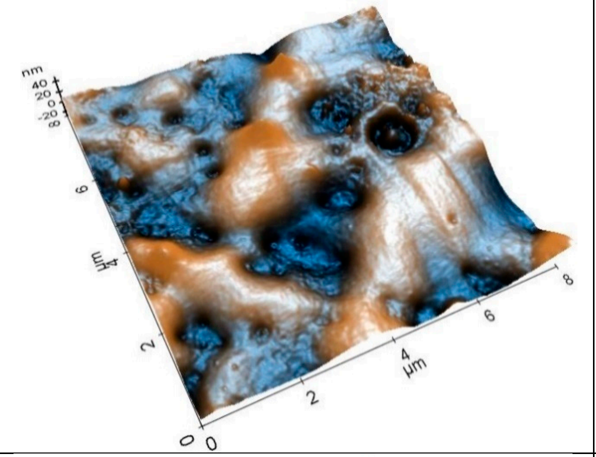	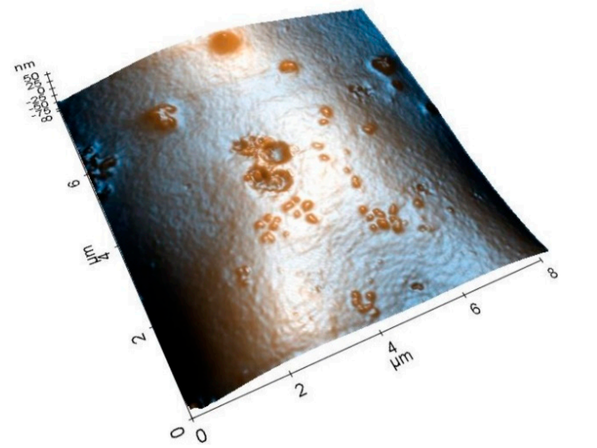
2 µm x 2 µm	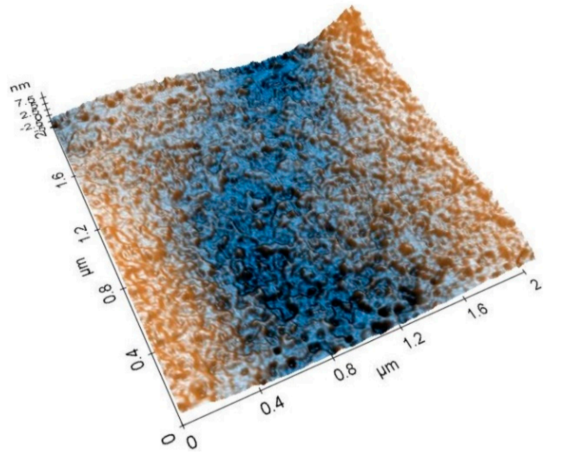	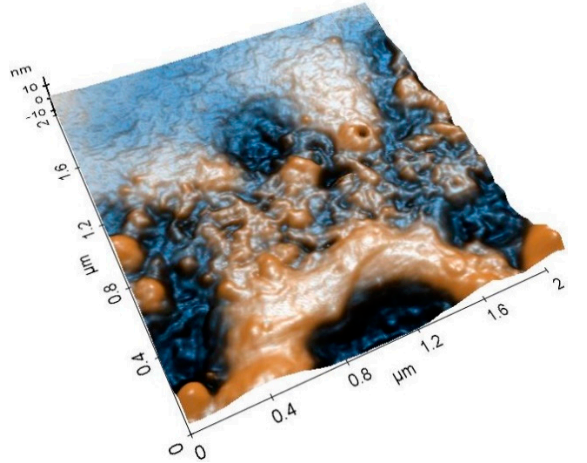	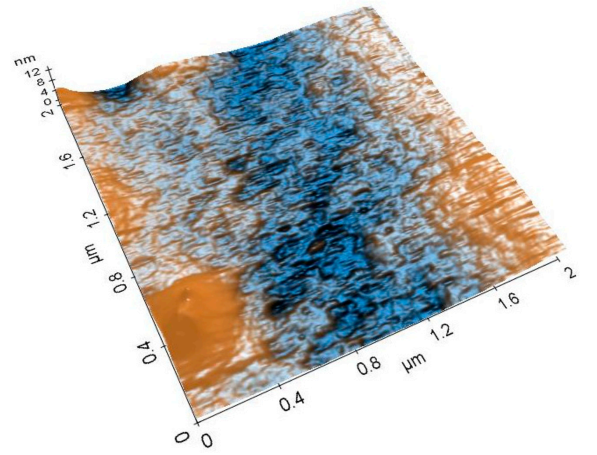

Figure S2. AFM 3D enhanced contrast images for ME samples (ME= *Meliloti herba* extract, PHB = polyhydroxybutyrate, PLGA = polylactic-co-glycolic acid).

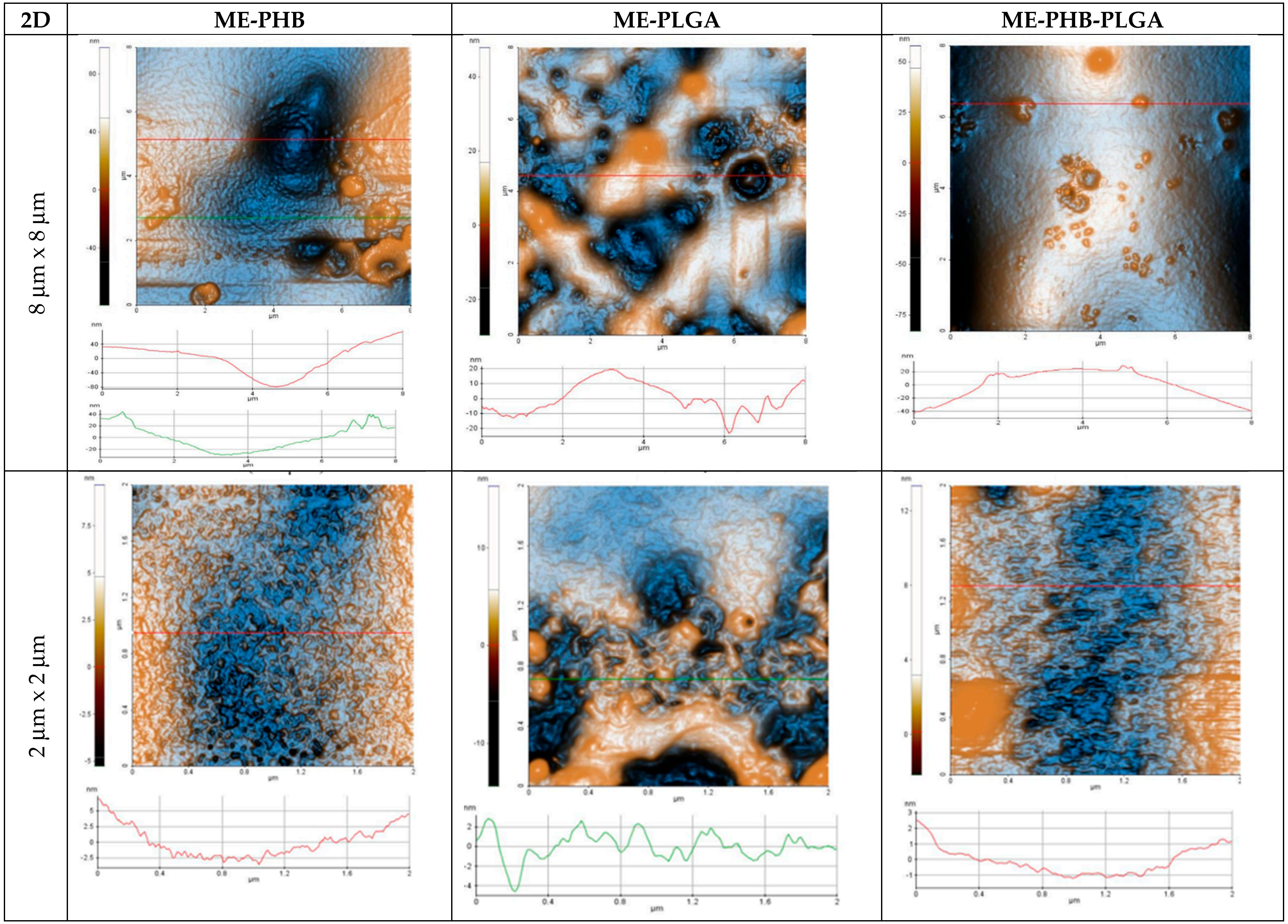

Figure S3. AFM 2D enhanced contrast images for ME samples with profile lines (ME= *Meliloti herba* extract, PHB = polyhydroxybutyrate, PLGA = polylactic-co-glycolic acid).

Table S2. Roughness parameters for ME samples (ME= *Meliloti herba* extract, PHB = polyhydroxybutyrate, PLGA = polylactic-co-glycolic acid, Rpv = peak to valley, Rq = root mean square roughness, Ra = average roughness)

**Scale**	**8×8 μm²**			**8×8 μm² profile line**			**2×2 μm²**			**2×2 μm² profile line**		
**Parameter (nm)** / **Sample**	**Rpv**	**Rq**	**Ra**	**Rpv**	**Rq**	**Ra**	**Rpv**	**Rq**	**Ra**	**Rpv**	**Rq**	**Ra**
EM-PHB	177.945	25.368	19.208	153.189	41.657	34.703	14.928	2.448	2.006	10.379	2.453	2.044
EM-PLGA	77.452	8.656	6.691	42.634	10.251	8.685	30.697	2.915	1.894	7.385	1.35	1.02
EM-PHB-PLGA	140.895	23.852	20.126	71.552	21.839	19.576	15.503	1.566	1.021	3.713	0.877	0.723

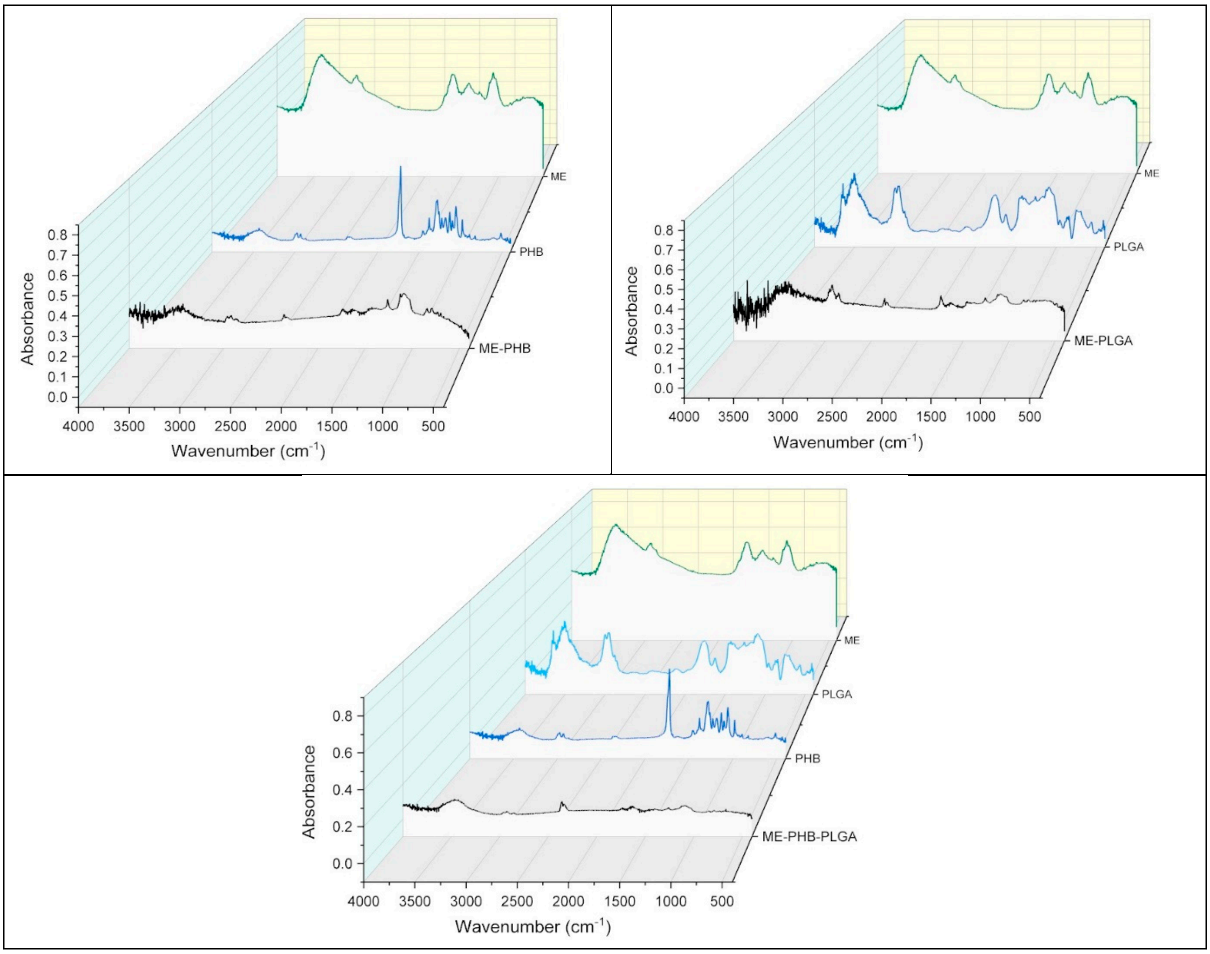

Figure S4. ATR-FTIR comparative spectrograms for ME samples (ME= *Meliloti herba* extract, PHB = polyhydroxybutyrate, PLGA = polylactic-co-glycolic acid).

**Table S3.** ATR-FTIR comparative analysis for ME samples (ME= *Meliloti herba* extract, PHB = polyhydroxybutyrate, PLGA = polylactic-co-glycolic acid).					
	**ME**	**PHB**	**ME-PHB**	**ME-PLGA**	**ME-PHB-PLGA**
4000-2500	3395.3 (O-H alcohol, stretching vibration, hydrogen bonds) 2926.6 (2935-2915 cm^{-1} C-H, methylene group, stretching vibration, asymmetric) 2844.3 (2850-2815 cm^{-1} C-H, methoxi methyleter, stretching vibration)	3430.1 (O-H alcohol, stretching vibration, hydrogen bonds) 2974.1 (C-H, stretching vibration) 2963.1 (2970-2950 cm^{-1} C-H, methyl group, stretching vibration assymetric) 2866.5 (2880-2860 cm^{-1} C-H, methyl group, stretching vibration symmetric)	3445.9 (O-H alcohol, stretching vibration, hydrogen bonds) 2952 (2970-2950 cm^{-1} C-H, methyl group, stretching vibration asymmetric) 2917.2 (2935-2915 cm^{-1} C-H, methylene group, stretching vibration, asymmetric) 2669.7 (~2660 cm^{-1} O-H acid)	3447.4 (O-H alcohol, stretching vibration, hydrogen bonds) 2952 (2970-2950 cm^{-1} C-H, methyl group, stretching vibration asymmetric) 2926.6 (2935-2915 cm^{-1} C-H, methylene group, stretching vibration, asymmetric) 2853.8 (2865-2845 cm^{-1} C-H, methylene group, stretching vibration symmetric)	3468.1 (3550-3450 cm^{-1} O-H alcohol, OH dimeric,stretchin vibration) 2955.1 (2935-2915 cm^{-1} C-H, methylene group, stretching vibration, asymmetric) 2926.6 (2935-2915 cm^{-1} C-H, methylene group, stretching vibration, asymmetric) 2847.5 (2865-2845 cm^{-1} C-H, methylene group, stretching vibration symmetric) 2845 (2850-2815 cm^{-1} C-H, methoxi methyleter, stretching vibration)
2000-1500	1625.3 (1680-1620 cm^{-1} C=C, alkenyl group, streching vibration)	1733 (1750-1725 cm^{-1} C-O ester 1740-1725 cm^{-1} C=O aldehyde (band in area 2800-2700 cm^{-1} stretching vibration of C-H for end-aldehyde))	1736.1 (1750-1725 cm^{-1} C-O ester 1740-1725 cm^{-1} C=O aldehyde (band in area 2800-2700 cm^{-1} stretching vibration of C-H for end-aldehyde))	1742.6 (1750-1725 cm^{-1} C-O ester 1740-1725 cm^{-1} C=O aldehyde (band in area 2800-2700 cm^{-1} stretching vibration of C-H for end-aldehyde)) 1641.2 (1680-1620 cm^{-1} C=C, alkenyl group, streching vibration)	1644.3 (1680-1620 cm^{-1} C=C, alkenyl group, streching vibration)

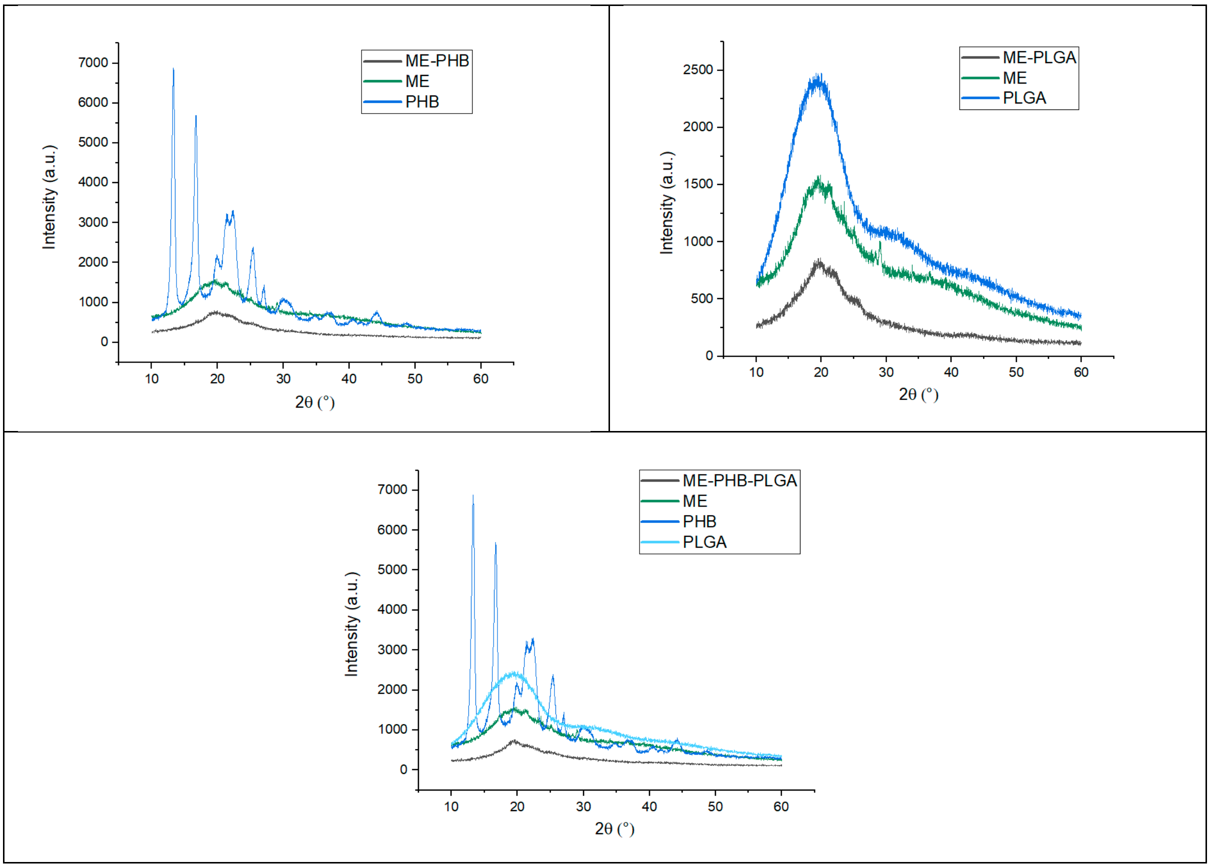

Figure S5. XRD comparative results for ME samples (ME= *Meliloti herba* extract, PHB = polyhydroxybutyrate, PLGA = polylactic-co-glycolic acid).

Table S4. The content of formulation impregnated on the compression stockings matrix estimated by thermogravimetry for ME samples (ME = *Meliloti herba* extract, PHB = polyhydroxybutyrate, PLGA = polylactic-co-glycolic acid, CS = compression stockings material).

Sample	Formulation (%)	Compression stockings material (%)
CS-ME-PHB	2.7	97.3
CS-ME-PLGA	10.8	89.2
CS-ME-PHB-PLGA	12.4	87.6

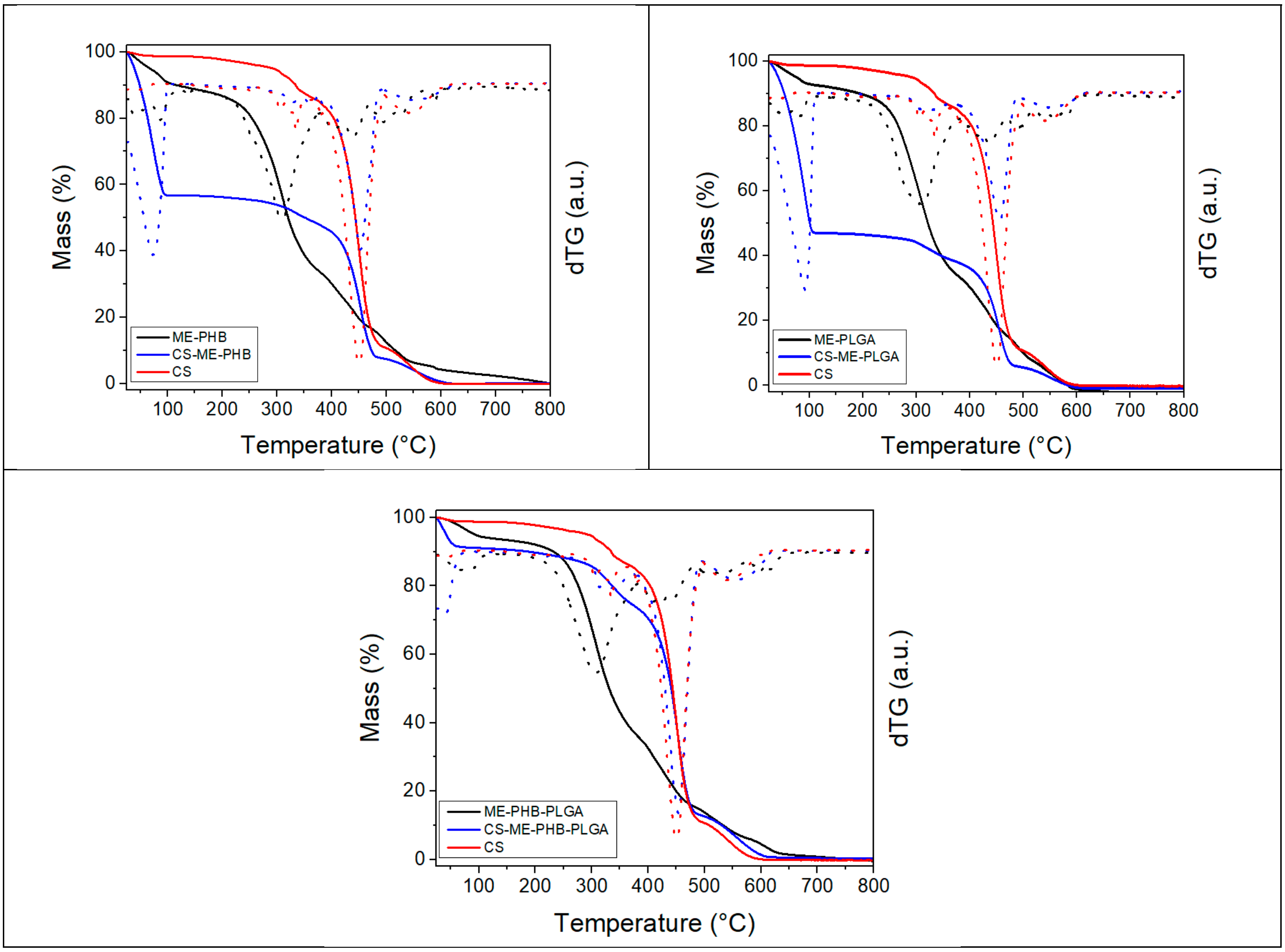

Figure S6. Thermogravimetric results for ME samples (ME= *Meliloti herba* extract, PHB = polyhydroxybutyrate, PLGA = polylactic-co-glycolic acid).
